# Information Technologies, Health, and Globalization: Anyone Excluded?

**DOI:** 10.2196/jmir.3.1.e11

**Published:** 2001-03-17

**Authors:** Florence Parent, Yves Coppieters, Marc Parent

**Affiliations:** ^1^Observatoire de la Santé du HainautHavréBelgium; ^2^School of Public HealthFree University of BrusselsBelgium; ^3^Institute of Tropical MedicineAntwerpBelgium

**Keywords:** Delivery of Health Care, Digital Divide, Information Inequality

## Abstract

Modern information technologies and worldwide communication through the Internet promise both universal access to information and the globalization of the medico-social network's modes of communication between doctors, laboratories, patients, and other players. The authors, specialists in public health and members of an association that aims to create opportunities for access to training in public health in developing countries, warn that the use of the term "globalization" ignores the reality of the "digital divide," that is, the fact that social inequalities may preclude the realization of this promise on a truly global scale.

## Information Technology, Globalization, and Social and Geographic Inequality

Two initial observations come to mind upon noting the facility with which the term "globalization" is applied to virtually everything: Enormous socio-economic differences exist, and it is known that the Internet network is mainly spreading in countries with a high gross domestic product and an open and competitive market in telecommunications. These distinctions are ignored when the term "globalization" is used.

Disease, risk behaviors (addiction to smoking, diet, sedentary life), and access to care are all correlated with socio-economic indicators such as family income, household composition, and parents' level of education [1,2]. It is a fact that we are not equal when it comes to prevalence of disease. Policy-makers and politicians working in the health field should set up adequate strategies in favor of populations at risk. The answer to inequality is "positive discrimination," that is, the answer lies in giving more means where needs are greater and, by doing so, decreasing iniquities in health. It seems that the development of information technology and improvements in health may create new needs. By answering to the exclusive demands of populations that can enter the market, these developments may increase inequality as well as reinforce the digital divide between industrialized countries and the developing world [3].

Let us take an example. According to the results of a national study on health among Belgians, single women with one or more children are one of the most vulnerable target groups. It seems unlikely that this group would have easy access to the Internet and all the new technologies linked with improving health. Moreover, it would be interesting to study the possible correlation between real access to and acceptability of such high technology tools on the one hand, and socio-economic factors on the other, especially in well-defined target groups. Differences in income and educational levels are the leading factors contributing to the divide in the United States [4]. The same approach could be used in relation to the development of technological devices aimed at improving patient home care.

Thus, the erroneous use of the term "globalization" in the context of information technology must be seen at the country and regional levels, but also at the supranational level. An example would be how difficult it is for associations and companies in the Southern Hemisphere to obtain commercial sponsoring for quality online services such as medical databases. Clearly, it wouldn't be opportune for sponsors to fund the development of high technology tools in an environment where the number of consumers with purchasing power is insufficient.

At this stage, one may question the usefulness of developing information technology in countries with a weak computer network. For example, there are currently more Internet users in New York City than on the entire African continent [5,6]. This question also applies to areas where there is sufficient accessibility for developing information technology for individual health and for the community (in order to differentiate the essential from the accessory in benefit). However, this question is even more pertinent for developing countries when resources are limited.

### Information Technology and the Need for Contextual Analysis

When one takes into account the actual benefits of efficacy and efficiency obtained through introducing information technology in increasingly global health strategies, it is necessary to recognize that the role of information technology is directly dependent on the context (the country and the health system).

#### Information Technology and the Health Information System

In countries with a high gross domestic product, improving the health information system through an Internet network means mainly improving exchanges between doctors and patients in the field of individual medicine. In Central and Western Africa, this type of tool could be aimed at increasing the efficiency of health systems in terms of statistical and epidemiological data collection as well as the ongoing establishment of indicators for running the different levels in the health system. Direct feedback at a decentralized level, such as at the level of the health district, is also a good tool for supervision and continuous evaluation. In these countries, the aim is mainly the improvement of strategies at the organizational and community levels.

#### Information Technology and Training

Another important field of use for multimedia tools is education and training. Indeed, there is a tendency to believe that use of the Internet necessarily means opportunities for long-distance learning (lectures and distance training); at-home, ongoing training; use of databases for clinical decision-making; and so on. But in countries where accessibility to the Internet or even to a computer is poor, it might not seem appropriate to propose training methods using information technology. However, to take this stance would mean that these tools would only be used as a mode of communication and information between individuals, with their great pedagogical potential forgotten. The use of new technologies is an important approach to teaching and learning [5] and, eventually, to quality training in countries where accessibility to such training is difficult.

The local context must be taken into account, and the development of this kind of tool at the central and regional levels as well as in training schools and universities must be promoted. It is mainly in these schools and institutions that computers are accessible and resources for computer maintenance are available. It is in these establishments, as well, that the training of trainers is most adequate and integrated into the national educational system. At this level, pilot projects can be undertaken and followed up in order to decentralize training and knowledge tools such as CD-ROMs. This mode of learning also gives students the opportunity to appropriate computer techniques included in the training.

In addition to the pedagogical benefits, the development of training projects with local schools reinforces their programs and their expertise and allows for the exchange of experiences and know-how between the Southern and the Northern Hemispheres. In the African context, for instance, the interest in using multimedia for training purposes lies more in the interactive potential and reinforcement of pedagogical means than in the setting up of a network. Of course, this use of information technology does not exclude, for instance, the development of Intranets or the Internet for telemedicine projects [6].

## Conclusions

It is important and strategically necessary both to be aware of the erroneous "globalization" concept and to recognize the numerous possibilities offered by information technology and multimedia in relation to their different contexts. It is even more important to realize that, in areas where there is no real market for the economy, it is still necessary and possible to apply information technology according to the needs of people and to utilize multimedia with well-defined objectives, for instance in community health and training.
